# First description of seasonality of birth and diagnosis amongst teenagers and young adults with cancer aged 15–24 years in England, 1996–2005

**DOI:** 10.1186/1471-2407-13-365

**Published:** 2013-07-31

**Authors:** Marlous van Laar, Sally E Kinsey, Susan V Picton, Richard G Feltbower

**Affiliations:** 1 Paediatric Epidemiology Group, Room 8.49 Worsley Building, Clarendon Way, University of Leeds, Leeds LS2 9JT, UK; 2 Paediatric Haematology, Leeds Institute of Molecular Medicine, University of Leeds, Leeds LS9 7TF, UK; 3 Regional Department of Paediatric Oncology and Haematology, Martin Wing, D Floor, Leeds General Infirmary, Great George Street, Leeds LS1 3EX, UK

**Keywords:** Cancer, Epidemiology, Seasonal, Adolescents

## Abstract

**Background:**

We aimed to examine evidence for an infectious aetiology among teenagers and young adults (TYA) by analysing monthly seasonality of diagnosis and birth amongst 15–24 year olds diagnosed with cancer in England.

**Methods:**

Cases of leukaemia, lymphoma and central nervous system (CNS) tumours were derived from the national TYA cancer register (1996–2005). Incidence rates (IR) and trends were assessed using Poisson regression. Seasonality of diagnosis and birth was assessed using Poisson and logistic regression respectively with cosine functions of varying periods.

**Results:**

There were 6251 cases diagnosed with leukaemia (n = 1299), lymphoma (n = 3070) and CNS tumours (n = 1882), the overall IR was 92 (95% CI 89–96) per 1,000,000 15–24 year olds per year.

There was significant evidence of seasonality around the time of diagnosis for Hodgkin’s lymphoma (*P* < 0.001) with a peak in February, and for ‘other CNS tumours’ (*P* = 0.010) with peaks in December and June. Birth peaks for those with ‘other Gliomas’ (Gliomas other than Astrocytoma and Ependymoma) were observed in May and November (*P* = 0.015).

**Conclusion:**

Our novel findings support an infectious aetiological hypothesis for certain subgroups of TYA cancer in England. Further work will examine correlation with specific infections occurring around the time of birth and diagnosis within certain diagnostic groups.

## Background

There is increasing evidence that environmental factors, such as infections, which vary seasonally and occur around the time of cancer diagnosis or around the time of birth, may affect subsequent development of cancer. This hypothesis can be tested by assessing temporal variation in cancer incidence as well as seasonality around the month of diagnosis and the month of birth amongst those with cancer; evidence for seasonality could reflect seasonal variation of infections.

Several studies worldwide have looked at seasonal patterns of cancer diagnosis. Summer peaks of ALL diagnoses have been observed amongst children and adults in East Anglia, [1] as well as for children across the UK, but only for diagnosis between 1953 and 1962. Summer peaks of ALL diagnoses as well as diagnoses of rhabdomyosarcoma and hepatoblastoma have been observed amongst children in the USA, and a winter peak for central nervous system (CNS) tumours diagnosed amongst children in southern USA [2]. Seasonal variation in month of diagnosis amongst children and adolescents for Hodgkin’s disease (HD) has been observed in Denmark with a peak in March [3]. Amongst adults, there is evidence of seasonality of diagnosis in England of monocytic leukaemia with a peak in February and March, and in Sweden melanoma diagnosis peak in May/June and September/October, prostate cancer diagnosis peak in October and breast cancer diagnosis peak in November [4,5].

Previous research has shown evidence for seasonality around the time of birth in relation to childhood cancer. For example, children diagnosed with acute lymphoblastic leukaemia aged 1–6 years in the North of England exhibited seasonality of birth, with peaks in February and March, [6,7] and a study from Hungary showed seasonality of birth amongst 0–4 year olds with ALL with peaks in February and August [8]. In contrast, several studies found no evidence of seasonality for ALL or by diagnostic group amongst children [9,10].

No studies have examined whether there is evidence of seasonality around the time of birth specifically amongst teenagers and young adults (TYA) with cancer, despite a number of papers studying these effects in childhood cancer. Although there are some studies which cover the childhood and adolescent age range for seasonality around the time of diagnosis, non focus solely on teenagers and young adults.

We examined evidence for any cyclical variation in temporal trends in TYA cancer incidence as well as monthly variation in months of diagnosis and birth, using a national cancer register of TYAs in England. We report findings from the first study to examine seasonality of cancer around birth and diagnosis specifically focused on the TYA age group, and as such we explored seasonal patterns in the three main tumour groups (leukaemia, lymphoma and CNS tumours) and their major subtypes for which evidence of seasonality already exists within the childhood cancer literature.

## Methods

### Data

Cases of leukaemia, lymphoma and central nervous system (CNS) tumours amongst 15–24 year olds diagnosed between 1996 and 2005 in England were obtained from the national TYA database. Data for this study were provided by the North West Cancer Intelligence Service following approval for the release of this information from the National Information Governance Board for Health and Social Care. Ethical approval for this study was obtained from Bradford Research Ethics Committee (Ref: 09/H1302/37). Yearly population data for 15–24 year olds by gender between 1996 and 2005 and month-specific birth populations per year from 1972 to 1990 were obtained from the office for national statistics. Birth populations by month and sex were not available, therefore, the distribution of males and females by year was used to estimate sex-specific birth population estimates for each month.

### Statistical methods

Incidence rates and trends were examined overall and by diagnostic subgroups. Incidence rates per 1,000,000 per year and 95% confidence intervals (CI) were calculated using yearly population figures from 1996–2005 and adjusted for sex. Incidence trends were assessed by deriving the average annual percentage change (AAPC) in incidence rates using sex-adjusted Poisson regression models, cyclical trends were assessed using Joinpoint regression analysis [11].

Seasonality of diagnosis was assessed by modelling the number of diagnoses by month using Poisson regression with the inclusion of cosine curves of 12 and 6 month periods (harmonic curves). Seasonality of birth was assessed by modelling the number of cases born in a certain month compared to the total monthly birth population at risk using logistic regression with the inclusion of harmonic curves of 12 and 6 month periods. As our data is restricted to both the age range at diagnosis and the year of diagnosis (15–24 year olds diagnosed between 1996 and 2005), the birth population at risk for each year is calculated as follows;

$$\begin{array}{l} {\mathrm{Birth}\;\mathrm{population}\;\mathrm{at}\;\mathrm{risk}}_{{\mathrm{month}}_{i}\;{,\mathrm{year}}_{j}}\\ \;=\left(\frac{\mathrm{Total}\;{\mathrm{births}}_{{\mathrm{month}}_{i}\;{,\mathrm{year}\;}_{{}_{j}}}}{n}\right)\;\times \;\left(\sum_{k=1}^{n}a_{k}\right) \end{array}$$

Where birth year *j* ranges from 1972–1990, *n* is the total number of diagnosis years (n = 10; 1996–2005) and α is the possible age for those born in year *j* who fall into the 15–24 age range between 1996 and 2005.

For example, a person born in January 1973 will be aged 23 in 1996 and 24 in 1997. Thus the birth population at risk in January 1973 is $\left(\frac{\mathrm{Birth}\;\mathrm{population}\;\mathrm{in}\;\mathrm{January}\;1973}{10}\right)\times \left(23+24\right)$.

Alongside goodness of fit statistics, we calculated Akaike’s information criterion (AIC) to determine the best fitting model in each case. The analysis was completed for all cases of leukaemia, lymphoma and central nervous system (CNS) tumours combined, as well as each major tumour group and subgroup as defined by the classification scheme for tumours diagnosed in adolescents and young adults [12]. Models were originally adjusted for sex, and subsequent analyses stratified by sex, allowing for varying seasonal patterns between groups. Seasonality was assessed by testing the significance of the peak(s) with the use of a likelihood ratio test, comparing models with a constant rate of cases per birth population at risk to the corresponding harmonic models for seasonality around the month of birth.

## Results

There were a total of 6251 cases of leukaemia, lymphoma and central nervous system tumours diagnosed amongst 15–24 year olds between 1996 and 2005 in England. Table 1 gives the incidence rates and average annual percentage changes by diagnostic group. The overall incidence rate (IR) is 92 (95%CI 89–96) per 1,000,000 per year. Lymphoma has the highest incidence amongst this age group (IR = 45; 95%CI 43–48 per 1,000,000 per year). There were no significant AAPC’s for any diagnostic group, except for a significant annual 6.82% increase amongst unspecified central nervous system tumours. No cyclical trends were observed between 1996 and 2005 in incidence overall or for any individual diagnostic group.

**Table 1:** Incidence rates and temporal trends for 15–24 year olds diagnosed with cancer in England, 1996-2005

		**Incidence rates**^**2**^		**Temporal trends**	
**Diagnostic group**^**1**^	**Cases ( *N *)**	**Rate**	**(95% CI)**	**AAPC**	**P-value**
Leukaemia	1299	17	(16–19)	0.12%	0.901
ALL	591	6	(6–7)	1.20%	0.401
AML	463	7	(6–8)	−1.47%	0.357
CML	147	2	(1–2)	4.98%	0.091
Other Leukaemia	98	1	(1–2)	−5.71%	0.095
Lymphoma	3070	45	(43–48)	1.17%	0.063
Hodgkin Lymphoma	2079	33	(31–35)	1.96%	0.078
Non-Hodgkin Lymphoma	991	12	(11–13)	0.79%	0.297
CNS Tumours	1882	29	(27–31)	0.37%	0.644
Astrocytoma	629	9	(8–10)	−0.58%	0.674
Other Gliomas	195	3	(2–3)	2.23%	0.374
Ependymoma	99	2	(1–2)	3.53%	0.321
Medulloblastoma	111	2	(1–2)	−2.68%	0.406
Other CNS	702	12	(11–14)	−0.50%	0.702
Unspecified CNS		2	(2–3)	6.82%	0.023
Leukaemia, Lymphoma, CNS tumours	6251	92	(89–96)	0.71%	0.106

^1^ Based on the classification scheme for tumours diagnosed in adolescents and young adults [12].

^2^Sex-adjusted rate per 1,000,000 per year.

*CI*: Confidence interval, *AAPC*: Average annual percentage change.

**Figure 1:**
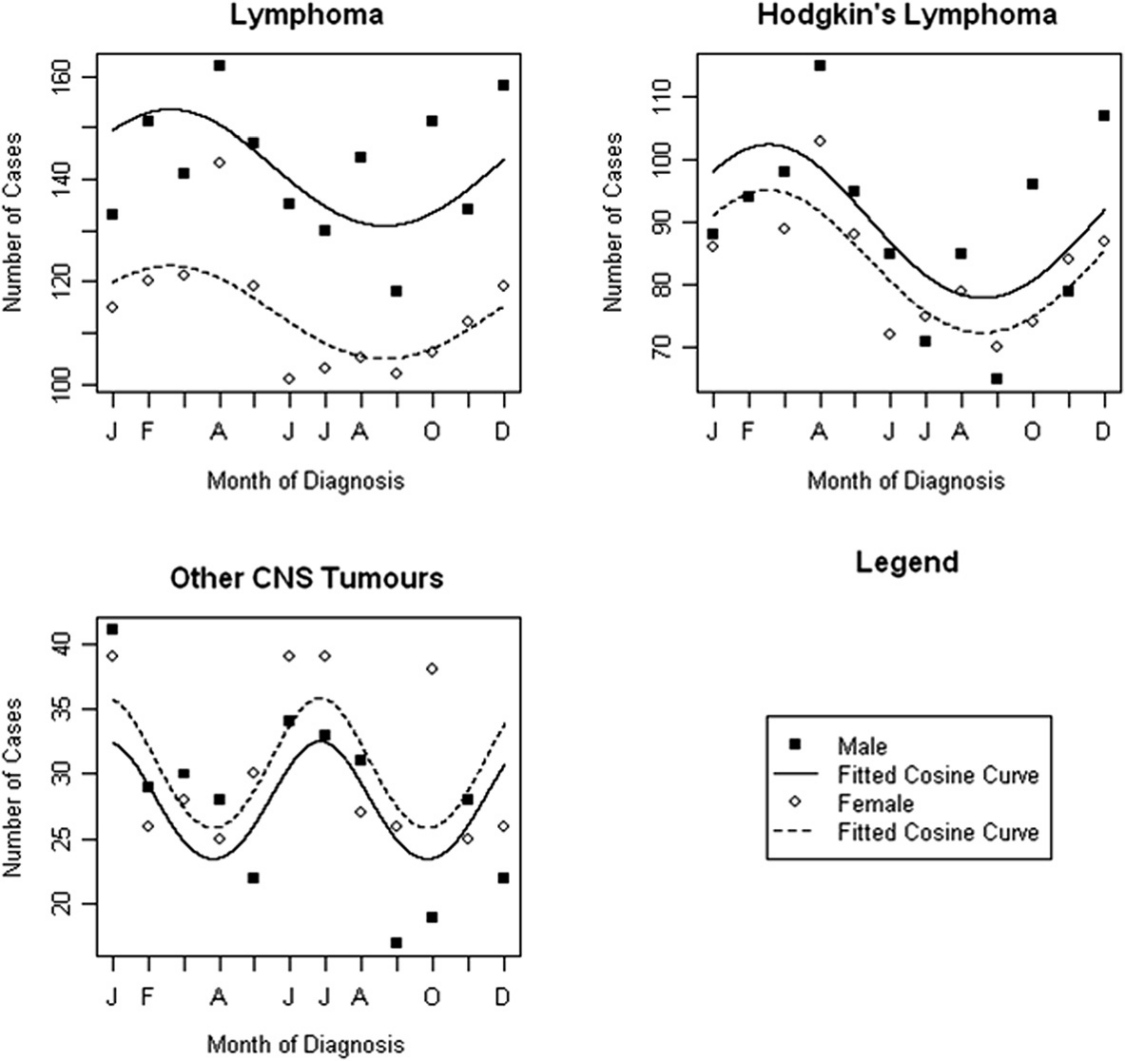
Sex-adjusted seasonality in month of diagnosis of cancer amongst 15–24 year olds, 1996–2005.

**Figure 2:**
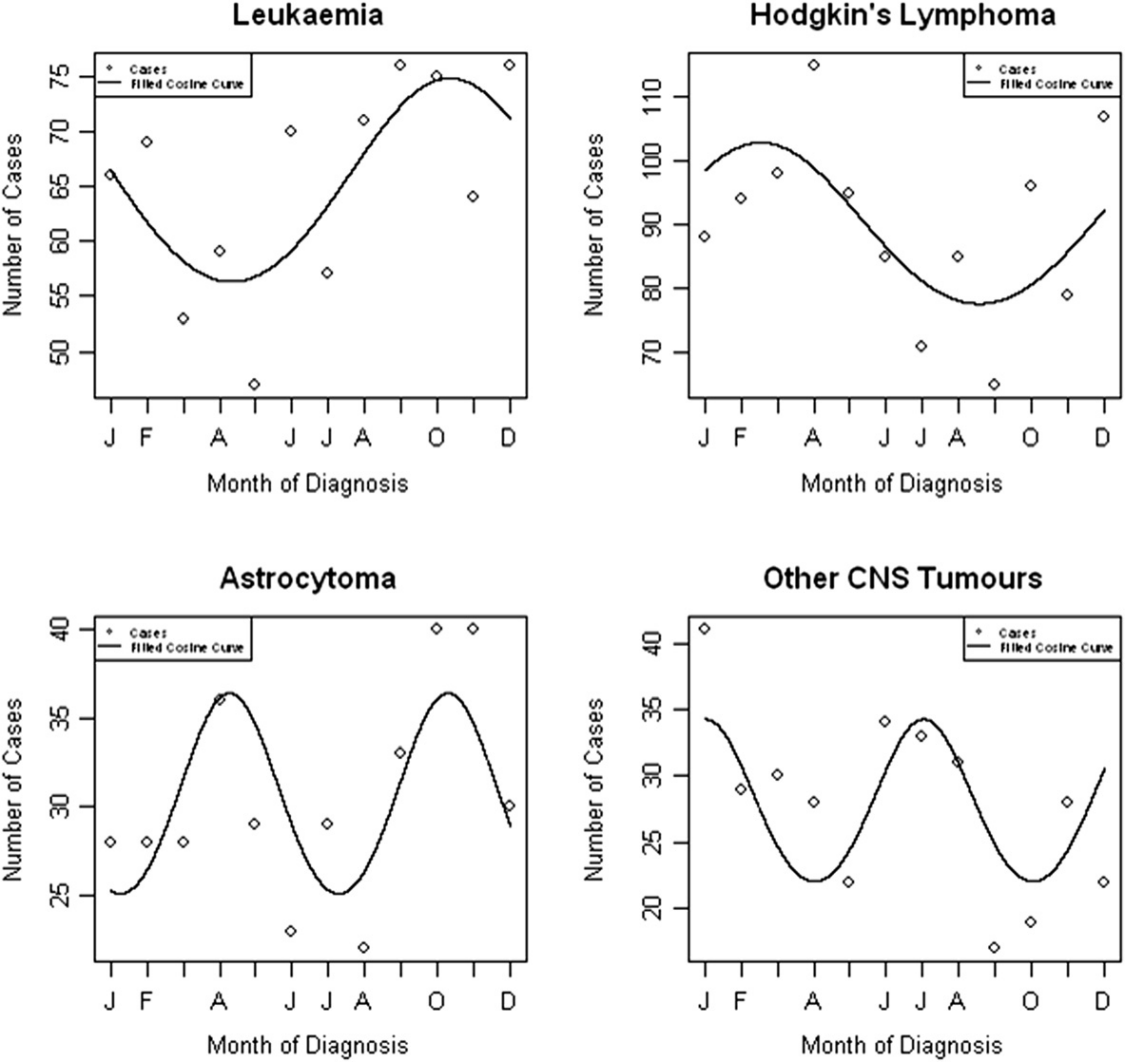
Seasonality in month of diagnosis for male 15–24 year olds with cancer, 1996–2005.

**Figure 3:**
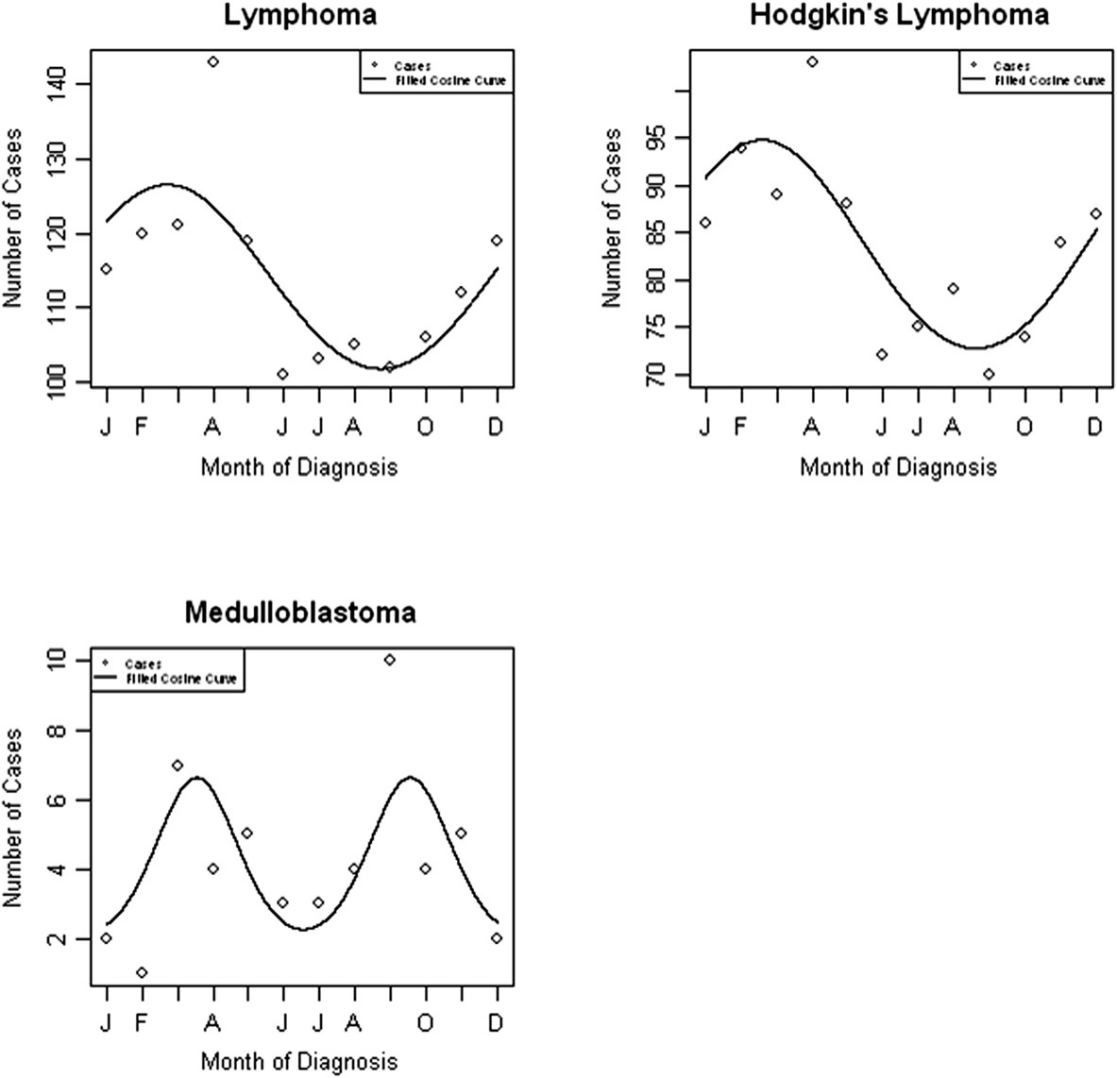
Seasonality in month of diagnosis for female 15–24 year olds with cancer, 1996–2005.

Table 2 gives the results of the Poisson regression models to assess seasonality around the time of cancer diagnosis. The best fitting model is given in each case, with either 12 or 6 month periods. We observed significant evidence of a 12 monthly seasonal effect in those diagnosed with lymphoma overall (*P* = 0.008) which was driven by Hodgkin’s lymphoma (*P* < 0.001) with peaks in February, and a 6 monthly seasonal effect in those diagnosed with other CNS tumours (*P* = 0.010) with peaks in December and June (Figure 1). The goodness of fit test gave *P*-values of 0.716, 0.580 and 0.305 respectively, indicating no evidence of any lack of model fit. When stratifying the analysis by sex, we observed significant seasonal effects of diagnosis with a 12 monthly cycle amongst males diagnosed with leukaemia (*P* = 0.020; peak in October) and Hodgkin’s lymphoma (*P* = 0.005; peak in February) and seasonality with a 6 month cycle for male diagnosis of astrocytoma (*P* = 0.043; peaks in April and October) and other CNS tumours (*P* = 0.018; peaks in January and July) (Figure 2). Amongst females, there was evidence of seasonality in month of diagnosis of lymphoma (*P* = 0.017; peak in February) and Hodgkin’s lymphoma (*P* = 0.013; peak in February), and evidence of a 6-monthly seasonal effect of medulloblastoma diagnoses (*P* = 0.032; peaks in March and September) (Figure 3). All goodness of fit statistics showed adequate model fit (*P* > 0.05)

**Table 2:** Sex-adjusted monthly seasonality of diagnosis in cancer amongst 15–24 year olds in England, 1996–2005

	**Harmonic model results**^**2**^					
**Diagnostic group**^**1**^	**Cases ( *N *)**	**Max**	**Min**	***P*-value**^**3**^	**GOF ( *P *-value)**	**Period (Months)**
Leukaemia	1299	Oct	Apr	0.266	0.461	12
ALL	591	Oct	Apr	0.253	0.780	12
AML	463	Nov	May	0.498	0.354	12
CML	147	Jan, Jul	Apr, Oct	0.651	0.690	6
Other Leukaemia	98	Feb, Aug	May, Nov	0.424	0.528	6
Lymphoma	3070	Feb	Aug	0.008	0.716	12
Hodgkin Lymphoma	2079	Feb	Aug	<0.001	0.580	12
Non-Hodgkin Lymphoma	991	Feb	Aug	0.667	0.968	12
CNS Tumours	1882	Sep	Mar	0.653	0.638	12
Astrocytoma	629	Dec, Jun	Mar, Sep	0.479	0.730	6
Other Gliomas	195	Sep	Mar	0.091	0.942	12
Ependymoma	99	Jan	Jul	0.612	0.988	12
Medulloblastoma	111	Dec, Jun	Mar, Sep	0.080	0.640	6
Other CNS	702	Dec, Jun	Mar, Sep	0.010	0.305	6
Unspecified CNS	146	May, Nov	Feb, Aug	0.448	0.657	6
Leukaemia, Lymphoma, CNS tumours	6251	Jan	Jul	0.241	0.793	12

^1^ Based on the classification scheme for tumours diagnosed in adolescents and young adults [12].

^2^Best fitting model is presented in each case, based on comparison of Akaike’s Information Criterion between models with differing periods.

^3^Statistical significance of the peaks.

*GOF*: Goodness of fit statistic.

**Figure 4:**
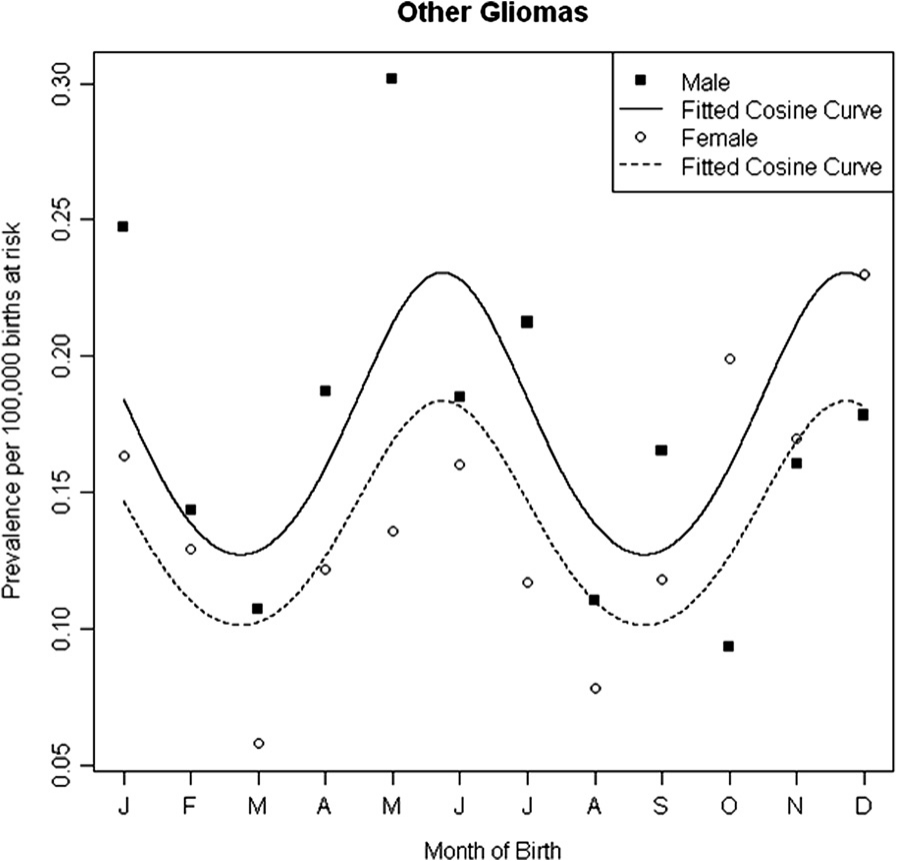
Sex-adjusted seasonality in month of birth amongst 15–24 year olds with Other Glioma’s, 1996–2005.

**Figure 5:**
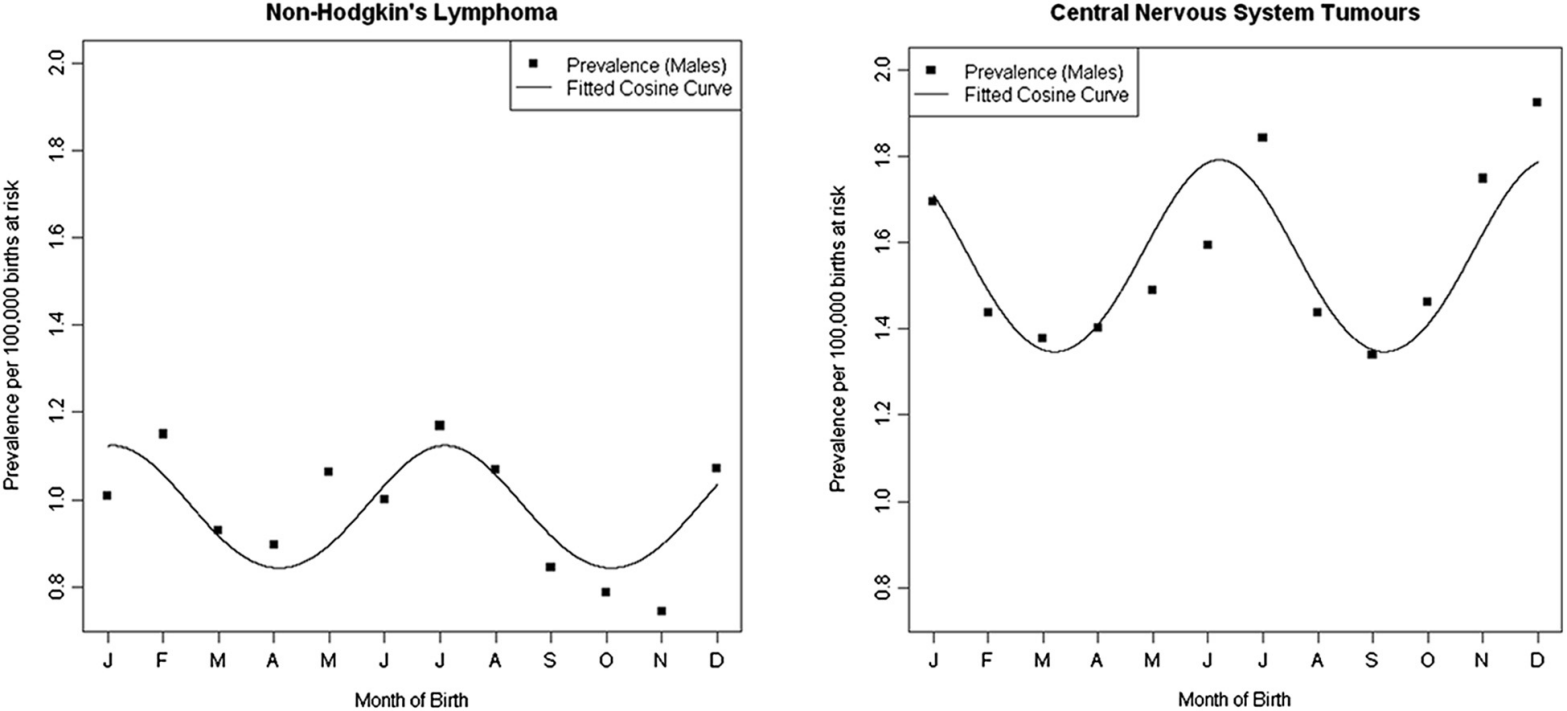
Seasonality in month of birth amongst male 15–24 year olds with Non-Hodgkin’s Lymphoma (left) and CNS tumours (right), 1996–2005.

Table 3 gives results of the sex adjusted logistic regression models including tests for seasonality of birth with 12 and 6 month periods. There was no evidence of seasonality with a 12 month period amongst any of the diagnostic groups. We observed significant evidence of a 6 monthly seasonal effect amongst those with ‘other Gliomas’ such that there were maximums in May and November and minimums in February and August (*P* = 0.015) (Figure 4). The goodness of fit test gives a *P*-value of 0.874, indicating good model fit. When stratifying the analysis by sex, we observed significant seasonal effects in males with non-Hodgkin’s lymphoma (peaks in January and July; *P* = 0.040) and CNS tumours (peaks in December and June; *P* = 0.006); no seasonality was present in females (Figure 5).

**Table 3:** Sex-adjusted monthly seasonality of birth in cancer amongst 15–24 year olds in England, 1996-2005

		**Harmonic Model Results**^**2**^				
**Diagnostic group**^**1**^	**Cases ( *N *)**	**Max**	**Min**	***P*-value**^**3**^	**GOF ( *P *-value)**	**Period (Months)**
Leukaemia	1299	Apr, Oct	Jan, Jul	0.242	0.917	6
ALL	591	Apr, Oct	Jan, Jul	0.242	0.917	6
AML	463	Jan, Jul	Apr, Oct	0.560	0.356	6
CML	147	Jan, Jul	Apr, Oct	0.775	0.650	12
Other Leukaemia	98	Jan	Jul	0.279	0.527	12
Lymphoma	3070	Jan, Jul	Apr, Oct	0.428	0.281	6
Hodgkin Lymphoma	2079	Mar	Sep	0.460	0.100	12
Non-Hodgkin Lymphoma	991	Jan, Jul	Apr, Oct	0.080	0.468	6
CNS Tumours	1882	May, Nov	Feb, Aug	0.155	0.368	6
Astrocytoma	629	Jan	Jul	0.119	0.988	12
Other Gliomas	195	May, Nov	Feb, Aug	0.015	0.874	6
Ependymoma	99	Dec, Jun	Mar, Sep	0.158	0.092	6
Medulloblastoma	111	Jan, Jul	Apr, Oct	0.257	0.446	6
Other CNS	702	Apr	Oct	0.706	0.116	12
Unspecified CNS	146	Dec	Jun	0.753	0.616	12
Leukaemia, Lymphoma, CNS tumours	6251	Jan	Jul	0.339	0.405	12

^1^Based on the classification scheme for tumours diagnosed in adolescents and young adults [12].

^2^Best fitting model is presented in each case, basedon comparison of Akaike’s Information Criterion between models with differing periods.

^3^Statistical significance of the peaks.

GOF: Goodness of fit statistic.

## Discussion

This is the first national population based analysis in England which focuses specifically on seasonality of birth and diagnosis amongst TYAs with cancer.

There is existing evidence of seasonality around the time of diagnosis in children with leukaemia, Hodgkin’s lymphoma and brain tumours as well as some evidence of seasonality of birth for childhood leukaemia. An infectious aetiology for childhood cancers has been found in some diagnostic groups. Our study supports an infectious aetiological hypothesis for certain subgroups of cancer in TYAs in England.

Our findings show significant evidence of seasonality by month of diagnosis such that TYAs with Hodgkin’s lymphoma were most likely to be diagnosed in February. This pattern was further confirmed in subgroup analysis by gender for both males and females, and is consistent with that of a previous study looking at both children and adolescents under the age of 20 in Denmark which showed a peak of Hodgkin’s Lymphoma in March [3]. Two further studies have shown a March peak of Hodgkin’s lymphoma diagnoses, [13,14] however, these studies cover all ages and ages 0–79 respectively and did not focus exclusively on the TYA age range.

Although no specific infectious agent has been identified as a cause for Hodgkin’s lymphoma, there have been numerous studies which have reported an association between Hodgkin’s lymphoma and Epstein-Barr virus (EBV). Mononucleosis is a viral infection usually caused by EBV, which has been shown to have similar seasonal patterns as that of Hodgkin’s lymphoma amongst those under the age of 40, with a peak in March [15].

TYAs diagnosed with tumours classified as ‘other CNS tumours’ displayed a significant winter and summer peak overall, which appeared to be driven by males. Winter peaks have been observed in previous childhood studies of CNS tumours, [2] but not in the TYA age range. The gender specific analysis further revealed significant seasonality of male diagnosis of astrocytoma peaking in April and October and female diagnosis of medulloblastoma peaking in March.

The bi-modal seasonal birth peaks observed in other CNS tumours (excluding Ependymomas and other Gliomas) and those amongst males with astrocytoma are a surprising result given the wide spectrum of clinical behaviours within both of these heterogeneous groups, which include both low and high grade brain tumours. Infections tend to display seasonal winter peaks, however, there may be a significant lag time between when the first cancer symptoms occurred and the date of diagnosis. Studies looking at the lag time for brain tumours in the TYA age range give inconsistent results, with a median reported delay of around 6 weeks in one paper [16] and a mean lag time of 13 weeks in another [17]. Potential confounding by delays in diagnosis therefore requires careful consideration when attempting to interpret these results. Nonetheless, this novel finding highlights the need for further aetiological research into CNS tumours and their subtypes, which are currently under-researched in this age group.

Additionally, we observed a peak in October for male diagnoses of leukaemia. Although childhood studies of seasonality in leukaemia have shown peaks of diagnosis in summer months, [2] another study for adults has shown they have peaks in winter months [4] which is similar to our finding. Differences between the seasonality amongst childhood leukaemia compared to TYA leukaemia are not necessarily unexpected, due to the rapidly progressive nature of leukaemia amongst TYAs compared to children and the changes in disease epidemiology and tumour biology such that the incidence of ALL decreases and AML increases when moving through the childhood and young adult age range.

In addition to seasonality around diagnosis, we also observed significant evidence of seasonality by month of birth. There were significant peaks in the birth months of May and November for males and females who developed ‘other Gliomas’ (Gliomas that are not classified as Astrocytoma or Ependymoma). This finding was difficult to interpret due to the heterogeneous nature of the ‘other Glioma’ classification containing both low grade Gliomas such as Oligodendroglioma and Oligoastrocytoma as well as aggressive high grade Gliomas such as Gliomatosis. Small numbers precluded any further analysis by diagnostic subtype. Further seasonal effects around birth were restricted to males with non-Hodgkin’s lymphoma and males with CNS tumours, both exhibiting peaks in mid-winter and mid-summer.

In terms of aetiology, our results suggest that exposures around the time of diagnosis are more important for TYA with cancer than environmental factors operating around the time of birth. This is contrary to epidemiological findings observed amongst childhood cancer diagnosed under the age of 15. This could be evidence for a more important role of environmental factors rather than innate factors alone, due to the increased time period between birth and diagnosis for TYAs compared to children. More generally, a viral infection of the mother transmitted in utero to the foetus may be the precipitating event leading to cancer in adolescence and young adulthood. This mechanism has been described for other chronic diseases including Type I diabetes [18,19] and celiac disease [20].

Evidence of seasonality around the month of diagnosis and month of birth could be explained by a number of factors; they could be a result of increased routine GP/health care appointments in winter leading to higher chances of diagnosis around this time; [21] or to seasonal differences in dietary consumption, [22] or a response to an infectious agent acting either around the time of diagnosis or around the time of birth [23] or they could simply be chance findings due to the number of statistical tests that have been performed. A limitation of the work is that the analysis did not include any data on perinatal factors which may influence the risk of cancer amongst TYAs. Nevertheless, the consistency with other studies provides greater confidence that these may be more than chance findings; furthermore, all models had good model fit statistics.

Population mobility is relatively high for individuals aged between 15–24, with many moving to college or University or entering employment for the first time. This may lead to increased exposure to unknown infections which may vary in ways which might explain the bi-modal seasonality of cancer diagnosis observed within this study.

## Conclusion

This is the first national population based study in England focusing specifically on seasonality of birth and diagnosis amongst TYAs with cancer. An infectious aetiology for childhood cancers has already been shown in some diagnostic groups, however, this study has produced new findings describing seasonality patterns around the time of diagnosis and birth amongst the TYA age group in England.

Our study supports an infectious aetiological hypothesis for certain subgroups of cancer in TYAs in England. We have shown seasonality in diagnosis of Hodgkin’s lymphoma and the other CNS tumour subgroup as well as seasonality in birth for the other glioma subgroup. This work forms the basis for further investigations into the infectious aetiology of TYA cancers by examining correlation with specific infections occurring around birth (including antenatal exposure to infectious or seasonal environmental factors) and diagnosis within certain cancer subtypes.

## Competing interests

The authors declare that they have no competing interests.

## Authors’ contributions

MvL carried out the data cleaning, statistical analysis and drafted the manuscript. SEK and SVP conceived the study and participated in the design and helped draft the manuscript. RGF participated in the design and coordination of the study, helped with the statistical analysis and to draft the manuscript. All authors read and approved the final manuscript.

## Pre-publication history

The pre-publication history for this paper can be accessed here:

http://www.biomedcentral.com/1471-2407/13/365/prepub
